# Ionizing Radiation and Human Health: Reviewing Models of Exposure and Mechanisms of Cellular Damage. An Epigenetic Perspective

**DOI:** 10.3390/ijerph15091971

**Published:** 2018-09-10

**Authors:** Ernesto Burgio, Prisco Piscitelli, Lucia Migliore

**Affiliations:** 1 European Cancer and Environment Research-ECERI, 1000 Bruxelles, Belgium; 2 Euro Mediterranean Scientific Biomedical Institute-ISBEM Research Centre, 72023 Mesagne (Brindisi), Italy; priscofreedom@hotmail.com; 3 Department of Translational Research and New Technologies in Medicine and Surgery, University of Pisa, 56126 Pisa, Italy; lucia.migliore@med.unipi.it

**Keywords:** ionizing radiations, cellular damage, carcinogenic mechanisms, epigenetic mechanisms

## Abstract

We reviewed available evidence in medical literature concerning experimental models of exposure to ionizing radiations (IR) and their mechanisms of producing damages on living organisms. The traditional model is based on the theory of “stochastic breakage” of one or both strands of the DNA double helix. According to this model, high doses may cause the breaks, potentially lethal to the cell by damaging both DNA strands, while low doses of IR would cause essentially single strands breaks, easily repairable, resulting in no permanent damages. The available evidence makes this classical model increasingly less acceptable, because the exposure to low doses of IR seems to have carcinogenic effects, even after years or decades, both in the exposed individuals and in subsequent generations. In addition, the cells that survived the exposure to low doses, despite being apparently normal, accumulate damages that become evident in their progeny, such as nonclonal chromosomal aberrations, which can be found even in cells not directly irradiated due to the exchange of molecular signals and complex tissue reactions involving neighboring or distant cells. For all these reasons, a paradigm shift is needed, based on evidence and epigenetics.

## 1. Introduction

The danger of ionizing radiations (IR) on human health is well known since the last century. There is a general agreement that high doses of IR represent a major threat to human health. At the opposite end of the spectrum, many scientists have expressed growing doubts and proposed different models concerning the risks linked to persistent exposures to small doses of ionizing radiations, which are much more frequent than accidental xposure to high doses. These potential risks could recognize new biological mechanisms of damage, including epigenetic, procarcinogenic pathways and transgenerational transmission. The adoption of the patterns of exposure, risk assessment, and damage (especially carcinogenicity) in environmental health (particularly IR) are inevitably affected by the way in which history determined and conditioned the research. 

It is for this reason that, to better understand the necessity of a paradigm shift, we need to start from a brief historical assessment of radiobiology, a discipline dominated by physicists who described for decades the interactions between radiations and living matter mainly in terms of energy transfers and DNA damage. In fact, radiobiologists focus on a passive, mechanistic model of DNA damage, even if emerging evidence in the field of molecular biology shows that the interactions between IR and living organisms, starting from the controversial issue of carcinogenesis [1], should be studied in a systemic way, taking into account the complexity of tissues, cell signaling and (epi)genetic reactions involved. 

The so-called linear and no-threshold model (LNT) has been recognized for half a century as the methodological basis for predicting long-term biological damage caused by IR. This model is still accepted by the most relevant international agencies and researchers. 

The second pillar of classical radiobiology arose from a more precise definition of the primary damage to DNA, which followed the description (in 1961) of stochastic breakage of one or both strands of the double helix (single-strand breaks (SSBs); double-strand breaks (DSBs)), interpreted as the primary lesions in DNA exposed to IR. On this basis, in 1973, the linear quadratic equation (LQ-Linear Quadratic equation) was formulated, based on the idea that low doses of ionizing radiation should essentially cause SSBs, easily repairable, while high doses would cause the breaks, potentially lethal to the cell, of both strands of the double helix of DNA (for low doses we mean, along the text, doses below 0.5 Gray). According to this model, only a massive exposure to IR (of the order of 1–2 Gray or more) could determine significant damages to tissues or human health, and the effects should be distinguished by deterministic (caused by direct cellular damages) and stochastic effects. The deterministic effects are almost immediate: the short-term exposure to massive doses of IR on proliferating tissues (bone marrow, blood, and epithelial cells in adult organisms; many different cell types in developing organisms) would cause the death of millions of directly affected cells. The effects should be directly proportional to the extent of the damage and the duration of the exposure: bone marrow aplasia, bleedings, blood poisoning, coma, and death could arise within minutes/hours from massive exposures to IR; anemia, aging, diarrhea could be induced by more diluted massive exposures. According to this model, also the stochastic effects would depend from the total dose of IR, and could cause—through the free radicals and reactive oxygen species (ROS) produced by the radiolysis of water—“stochastic” damages on DNA resulting in procarcinogenic effects if the affected genes are involved in cell cycle control or in programmed cell death (apoptosis) and DNA repair (proto-oncogenes and tumor suppressor genes).

The great majority of epidemiological data concerning the effects of exposures to IR came from the cohorts of the survivors of the bombing of Hiroshima and Nagasaki and the fallout of radionuclides after the Chernobyl accident and, to a lesser extent, from the studies on the effects of prolonged X-ray occupational exposures (i.e., in medical diagnostics): very different situations, characterized by totally dissimilar modes and types of exposure [2]. Nevertheless, the modalities of exposure assessment, damage (especially carcinogenicity), and radioprotection have been heavily influenced by these theoretical models.

In the case of Hiroshima and Nagasaki survivors, a hundred thousand people were subject to a massive total body short-term exposure to large doses of radiation at low and high LET (linear energy transfer). The results of such exposure were the almost instantaneous death of thousands of women, men and children; the slow agony of several thousand victims whose tissues had been irreversibly damaged by β particles and neutrons produced by the explosion; a great number of diseases (neoplastic or not) observed after years or decades both among survivors and their offspring. As for cancer the first relevant data was a significant increase of leukemias (excluding chronic lymphatic leukemia), recorded in the years immediately following the bombing [3]. After some years, the epidemiologists detected a significant increase of solid tumors in various tissues and organs, especially thyroid, breast, and lung cancer [4,5,6]. However, important limitations concerning these studies have been reported: the count of the survivors was not accurate, and the data began to be reliable only many years after the exposure but are still incomplete [7]. Another critical note concerns the thousands of dramatic events (abortions and deaths of children), that occurred in the first weeks/months after the tragedy that have never been recorded. Yet, the most difficult fact to explain concerned the offspring of the survivors, as most studies did not record in the children born from the more exposed parents, especially in the early decades [8], neither an increase of mutations, [9] nor an excess of congenital malformations [10], nor a significant increase in tumors [11]. Only many years later, based on more sophisticated investigations, was it possible to clarify the significance of these apparently reassuring and paradoxical data [12]. Indeed, a short-term exposure to massive doses of IR, resulting in cellular irreversible damage, determined a twofold effect of selection: first on cells and tissues of the survivors, and then on the general population (as the survivors are, by definition, a selected component of the entire exposed population).

The limited total count of harmful effects in a population exposed to high total doses of IR and the supposed absence of major differences between more or less exposed subjects led the majority of researchers and international agencies to underestimate for decades the risks of radiations, especially regarding the effects of prolonged exposure to low doses, which are the most frequent and most dangerous for human being. This serious error of judgment had its roots in the way in which the studies were conducted based on the LNT model and considering the total absorbed doses on the basis of the distance from the epicenter of the explosion, through extremely complex calculations, revised several times [13].

## 2. Increasing Evidence Fostering a Paradigm Change

The first criticisms to the LNT model had already emerged in the 50s, when researchers tried to experimentally prove its limits by analyzing the studies concerning genetic damages induced by IR [14]. More specific criticisms emerged when scientists began to understand that the target of IR was not only the DNA, but the entire cell (*membrane theory*) [15]. However, almost all the experts and researchers uncritically accepted the basic concept of the LNT model: the dependence of the damage from the total dose. 

One of the biggest mistakes in the studies carried out during the 60s and 70s was the assumption that an irradiated cell that survives and is still able to replicate should be considered as healthy as a never irradiated cell. Most scientists tried, by this way, to preserve the concept of the importance of the initial stochastic damage of DNA, excluding the possibility that the damaging effects suffered by cells (and DNA) could slowly accumulate over the time and that such a cumulative damages effect could occur even after months or years. The awareness has therefore begun to emerge that the cells that survived after exposure to IR, despite being apparently normal, could accumulate damages. In fact, scientists found that the irradiated cells could not go beyond the 7–10 divisions (instead of the normal 60–70) and, above all, that some mutations and lethal events become evident only in the progeny of irradiated cells [16]. Some researchers began to theorize the progressive accumulation of mutations and chromosomal aberrations and to envisage the idea of critically rethinking the thesis, utterly implausible, of the absolute normality of the irradiated survived cells [17].

An important turning point occurred in 1992, when a study documented the occurrence of nonclonal chromosomal aberrations in the progeny of bone marrow cells irradiated by α particles [18]. For the traditional radiobiology it was a real heresy, as in the dominant model the genetic damage is directly caused by the radiation and therefore precocious, while—according to this study—the damage seemed to consist mainly in a progressive genomic instability affecting the different cell populations of the irradiated marrow. The most likely explanation for this unexpected phenomenon was that IR caused the pathological activation of bone marrow stem cells (poorly differentiated), which produced, in the subsequent cell generations, genomic alterations.

In subsequent years, experimental studies in hematology, which tried to expose cells characterized by different degrees of differentiation to various types and doses of IR, proved that cultures of irradiated bone marrow cells showed more evident occurrence of secondary genomic instability, which seemed to play a key role in the lymphoma-leukemogenesis due to IR [19]. Another important step was made when it was shown that cells of murine strains—that did not develop leukemia from radiation exposure—were resistant in vitro and did not develop neither genomic instability, nor subsequent chromosomal aberrations [20]. Some years later the existence of similar mechanisms of genomic instability induced in vivo by long-term small doses of IR began to be demonstrated also in humans [21].

Also, the classical model of DBSs induced by radiations in proportion to their energy proved to be increasingly less acceptable, as the supposed clonal mutations, which would have demonstrated the critical role of the primary event, were not regularly present in the progeny of irradiated cells, while there were diverse chromosomal aberrations due to chromosomal and genomic instability [22]. The plausibility to connect dose and effect in a simple way vanished: it was increasingly clear that the initial irradiation triggered a slow reactive process and that damage and instability accumulated in the irradiated offspring. If mutations and chromosomal aberrations—far from being the primary event—represented late events, it was nonsense to propose a linear link between the initial dose and the biological damage [23].

However, the essence of this still ongoing conceptual revolution remained for a long time the prerogative of a few prominent experimental hematologists. In the following years, several studies were produced trying to make the point and to understand in what sense the new concept of genomic and chromosomal instability could change the entire representation of the radiation damage [24]. Those who wanted to preserve the old paradigm tried to use theories, such as that of the mutator phenotype [25], which were spreading in those years, proposing that—at the origin of genomic instability and chromosomal aberrations in cancerous cells—there were essentially stochastic mutations on specific preservative genes (tumor suppressor genes) or innate defects in DNA repair mechanisms. Yet, the evidence was other: genomic instability was current in many cells, not directly affected by radiations; not clonal mutations in the progeny were far more numerous than the clonal ones; minimal doses of radiation (about 2 mGy) were often sufficient to produce these effects [23,26,27]. At that point, it was more and more urgent to replace the deterministic model in which the damage was conceived as the effect of the energy associated with radiation (essentially inducing stochastic mutations in the DNA molecule) with a systemic model in which tissues and cells and the (epi)genome itself respond actively to damage. 

A further step forward was made by providing a better definition of the bystander effect. The fact that irradiation could involve cells not directly affected had already been reported in the 50s [28]. At the beginning of the 90s, it was demonstrated that in cells not directly affected by radiation—but close to the irradiated cells—mutations, micronuclei formation, chromosome breaks, and sister chromatid exchanges could occur [29]. It was soon realized that these were not isolated and random events, but secondary effects to complex tissue reactions and that in irradiated tissues the neighboring cells—even the distant ones from those directly affected—exchange molecular signals of harm and danger [30]. Among the molecular mediators involved, not only oxygen radicals (directly produced by the radiolysis of water), but also nitrogen radicals and cytokines were identified [31]. This also proved that a prolonged exposure to low doses of ionizing radiations can induce a low grade, systemic inflammatory response in the affected organism [32].

The fact that genetic damage, or even cell death, could affect cells not directly hit by radiations was an amazing event, which contradicted the tenets of the classical model of direct action of radiation on DNA (i.e., the immediacy and linearity of the dose-effect-damage). Other studies added interesting data, showing that subjects who received an organ transplant from irradiated individuals, could bear chromosomal aberrations in their cells, secondary to the presence of clastogenic factors in the donor [33], which, in hindsight, was not so new and shocking, as since the 60s clastogenic molecules were found in the blood of people exposed to ionizing radiation: in patients receiving prolonged radiotherapy as well as in the survivors of Hiroshima and Nagasaki [34] and, later on, in the liquidators and in children of Chernobyl [35]. The true, amazing novelty was that such substances coincided with the mediators of inflammation and oxidative stress involved in the bystander effect.

These studies demonstrated that the mechanisms of damage from exposure to low doses of radiation were fundamentally different from those at high doses. Above all, it became increasingly obvious that the effects of persistent exposures to low doses were potentially far more dangerous (in the medium and long term and at collective level) than the short-term exposure to massive doses: high doses produced damage consistent with the LNT model, and were lethal (and therefore selective!) for a significant proportion of cells, while the effects of exposure to small doses accumulated for years were causing tissue and systemic reactions and, most importantly, they were also inducing—both in the affected cells, in those not directly exposed and in their offspring—a progressive genomic instability, usually preceding the genetic mutations and chromosomal aberrations. Exposure to low doses of IR seemed to have carcinogenic effects, both in vitro [36] and in vivo [37], and chromosomal instability and tumors would be promoted by DNA hypomethylation [38]. 

## 3. The Chernobyl Lessons

Another confirmation of the necessity of a new model of exposure and damage due to IR arrived from Chernobyl studies [39]. The accident occurred in the Chernobyl nuclear power plant on 26 April 1986, causing the release of radioactivity into the atmosphere estimated at more than 1019 Bq (10,000 petaBq), with high levels of fallout in Belarus, Ukraine, Russian Federation and, although with noteworthy differences, over the entirety of Europe. The amount of people exposed to substantial levels of fallout was probably over 20 million in addition to the hundreds of workers who operated for two weeks to cover the molten core from above who were directly exposed to total body irradiation. Even today, after more than 30 years, the reconstruction of what really happened is difficult [40].

A first lesson came from the remarkable diversity of diseases caused compared with the cohort of the Hiroshima and Nagasaki survivors. Indeed, the fundamental difference concerned the nature of exposure: the population of the Japanese cities had been externally invested by a huge amount of high-LET neutrons and low-LET radiations, which had dramatically damaged tissues, cells and DNA. After the Chernobyl accident, huge amounts of radioisotopes concentrated in the food chains, thus resulting in a persistent internal irradiation. The short-term epidemiological consequences were a clear proof of the difference between the two situations: while in the first years after the atomic bombing the Hiroshima and Nagasaki survivors showed a significant increase of leukemias [3] and in the following decades an increase in solid tumors [5], in the case of Chernobyl (at least at that early stage) almost exclusively an organ, the thyroid, was affected, for its ability to actively capture and concentrate iodine (the amount of ^131^I in thyroid was about 1000–2000 times higher than that of the rest of the body) [41]. By the early 90s, the most significant health data concerned the increase of thyroid cancers (of a single type) in a growing number of children. The first official reports recorded 114 cases of thyroid cancer in Belarus children over a period of 30 months between 1990 and 1992, less than four years after the accident [42]. Moreover, they were overt cancers, which already showed direct invasion of adjacent tissues in 42% of children and metastasis in 6% [43]. To better evaluate these data we should consider that child cancers in Belarus had been 2 in 1986, 6 in 1989, and more than 100 in the following three years. Then, the epidemiological increase was exponential: in 1999 the cases in Belarus were 583, in Ukraine 324, and the incidence was about 10 times higher in the most exposed areas. If before the disaster the incidence of thyroid cancer was approximately 1 per million children each year, in 1995 the incidence had increased by 30 times in Belarus and by 100 times in the areas close to Chernobyl. By 1998 thyroid cancer incidence resulted significantly higher in children who were <2 age at the time of the explosion, which was obviously connected with the already known higher sensitivity of infants (and embryos) to radiation. Furthermore, the increase was greater in males, normally less subject than females to this type of cancer. At very high doses the risk of cancer decreased, while the risk of hypothyroidism increased, due to the destruction of the thyroid tissue [44]. 

Also, the histological, cytological, and molecular data proved to be extremely significant. The prevalent one was a specific form of cancer, in 98% of cases a papillary carcinoma, characterized by an unusual density of the neoplastic tissue [45]. In almost all cases a specific oncogene (*c-RET*, frequently implicated in papillary thyroid cancer [46]) was involved in translocations, better explicable as reactive-adaptive to ionizing radiations [45] than as stochastic mutations (as confirmed by in vitro studies [47]). 

Not only the International Atomic Energy Agency (IAEA) but also scientists were initially skeptical, both because it was thought, on the basis of the dominant model, that the isotopes released (^131^I, ^132^Te) representing the fundamental component of the fallout were not sufficiently carcinogenic, and because of the shortness of the latency time between the exposure and the increase of cancer. Today, the picture is clear: hundreds of cases of only one type of cancer are a unique event, from which we can draw some valuable lessons not only with regard to radiation damage, but also about the same modalities of reaction to radiations of the human genome.

If during the first years after the accident the I^131^ had the far greater pathogenic and carcinogenic role, today we can see the effects due to the permanence in the environment and food chains of other isotopes with much longer half-life—^134^Cs, ^137^Cs and ^90^Sr—to which may be ascribed the emerging problem of the increase in childhood leukemia, which was slower and not admitted by everybody. Once again, the disagreement is due to the different assessments of the exposure and the absorbed dose which, if calculated considering the current model, would be quite insufficient to cause increases in leukemia and other reported diseases. In fact, according to the official figures the effective dose of radiation absorbed by the fetus would have been about 2 mSv in Belarus and <1 mSv in the rest of Europe (0.02 mSv in the UK, 0.07 in Germany, 0.2 in Greece) [48], an exposure which according to the International Commission on Radiological Protection (ICRP) risk model would have an effect quite irrelevant. Instead, the data concerning a large population of over 2 million children exposed in utero after Chernobyl, not only in Belarus and Greece, where the exposure was more consistent [49], but also in Scotland, Wales, and Germany, where the exposure had been much lower [50,51] have recently led some researchers to calculate an increase of over 40% of cases of leukemia in children born in the period of the maximum peak of cesium in food (between 1 July 1986 and 31 December 1987), rather than in children born before the accident (between 1 December 1980 and 31 December 1985) or during the two following years (31 December 1985—1 January 1988) [52]. These data are highly significant and perfectly coincident with the cohort identified based on the data of exposure to ^137^Cs.

Above all, once again, the limits of the ICRP evaluation model were clear. Considering that the average dose of radiation absorbed during the embryo-fetal period by the cohort of the observed children (calculated on the basis of the external dose absorbed) was of 0.067 mSv, it was calculated that the use of the ICRP model would lead to an underestimation of the risk of about 100–160 times [53], which showed, in vivo, how little sense it makes to use a model conceived for evaluating short-term exposures to massive doses (which can induce a dramatic selection of cells and organisms), in situations of exposure to small daily doses of radiation, that may induce in the human genome (and in particular in the fetal genome) conditions of persistent and procarcinogenic genomic instability. 

The third lesson from the Chernobyl drama is in perspective the most important one, concerning the most feared effect of persistent exposure of entire populations to small doses of ionizing radiation: the (epi)genetic damage of human gametes and of subsequent generations and the transgenerational carcinogenesis, theorized for decades, but until then confirmed only in exceptional cases [54] and apparently excluded by the studies on the survivors of Hiroshima and Nagasaki [55]. 

In addition to the already mentioned incidence of thyroid cancers in children not directly exposed, as a result of a parental exposure; i.e., an exposure of the gametes—further evidence came from the observation that in children exposed to doses below 2 mSv and in the offspring of the liquidators high rates of microsatellite mutations were frequently observed [56]. An indirect, paradigmatic sign of the progressive genomic instability, which was found to be the main effect of a persistent exposure to low doses of radiation (and not of short-term exposures to massive doses, which have a powerful selection action also on the affected gametes). 

In order to better understand the real meaning of these effects, however, it is necessary to introduce some key concepts that have become more and more important in molecular biology during the last decade.

## 4. Genomic Instability: Towards an Epigenetic Paradigm of Ionizing Radiation Damage

It has been demonstrated, in recent years that the key mechanisms of genomic instability, in the cells and gametes affected by ionizing radiation, are both partial and global changes in DNA methylation. It is easy to understand the importance of studies demonstrating that the main molecular mechanisms involved in the genomic changes induced by exposure to small doses both in stem cells (with a possible procarcinogenic effect) and in germ cells (with transmission and amplification of a transgenerational damage) are essentially epigenetic and affect, in particular, the enzymatic mechanisms of DNA methylation.

The first studies showing the effects of IR on the epigenome date back to about twenty years ago: they documented a hypomethylation of the main sequence of DNA [57,58], with destabilizing and potentially procancerogenic effects on the genome [59,60]. Subsequent studies have compared the acute and chronic effects of low-dose radiations on DNA methylation: it was possible to analyze the different epigenetic modifications induced in various organs and tissues and, above all, to demonstrate that a chronic exposure to low doses of radiation represents a more powerful inducer of epigenetic modifications than acute exposures [61].

Another extremely significant piece of information that emerged from this research was that the epigenetic changes induced by IR sometimes are more persistent than genetic mutations [62]. Another study confirmed this paradoxical result, showing that most of the DSBs induced by IR, a month after exposure, had been repaired, while some epigenetic modifications (the hypomethylation of DNA) were more persistent and could play an important role in lymphoma-leukemo-genesis [63]. 

An increasing number of studies support the induction of alterations in global DNA methylation patterns and hypomethylation at specific cancer-related genes following low dose IR exposure [64,65]. For instance, an investigation on global hypomethylation and promoter hypermethylation of particular genes induced in mice by low dose IR (6 MV X-ray for 10 days; 0.05 Gy/day) was performed. The results showed that although IR induced genomic hypomethylation in blood 2 h postirradiation was not retained at 1-month, some genes displayed tissue-specific hypermethylation and downregulation, which persisted for 1-month postirradiation. A total of 811 genes were found to show specific hypermethylation, involving almost all the main biological processes, among which eight candidate genes (Rad23b, Tdg, Ccnd1, Ddit3, Llgl1, Rasl11a, Tbx2, and Slc6a15) were confirmed to be hypermethylated in low dose radiation samples [66].

Furthermore, transposable elements, that have been recently proved to be the key sensors and modifiers of DNA expression [67] appear also to be among the most sensitive genomic targets for agents able to induce epigenetic effects (including IR). Their activity is regulated by epigenetic mechanisms, including methylation of DNA and histone modifications. The loss of epigenetic control mainly consists in the loss of DNA methylation and changes in the chromatin structure resulting in transposable element reactivation (retrotransposition) and insertional mutagenesis. It has been reported in numerous human diseases, including cancer [68]. Although no data exist, to our knowledge, about the effects of exposure to IR on transposable elements (methylation, retrotransposition) in humans in vivo, some interesting findings have been obtained on human cell lines: indeed, both low- and high-LET irradiation may induce L1 retrotransposition in vitro, even if radiation quality-dependent differences were noted [69,70]. To evaluate the possible correlations between altered epigenetic profiles and genome instability, DNA methylation, mRNA, and miR levels were measured in individual cells clonally expanded (well characterized by their chromosomal stability/instability), exposed to both low linear energy transfer (LET) X-irradiation and high LET iron (Fe) ion irradiation. The results demonstrated correlations between epigenetic changes and IR- induced genomic instability [71]. Long Interspersed Nucleotide Element 1 (LINE-1) retrotransposons are the major repetitive elements in mammalian genomes, usually being heavily methylated. Studies of the group of Koturbash reported in mice global and LINE-1-associated DNA hypermethylation after exposure to various types of IR [68,72]. Exposure to ^56^Fe heavy ions increased the incidence of acute myeloid leukemia (AML) and the underlying molecular mechanisms seemed to be epigenetic alterations involving methylation of DNA and the expression of repetitive elements. These findings suggest that epigenetic reprogramming is involved in the development of IR-induced genomic instability and may have a causative role in the development of AML [73]. 

As for the transgenerational effects, the most interesting results came from studies that found specific epigenetic marks in the offspring of exposed subjects. We have already mentioned that various types of IR can induce a state of genetic instability in the germ cells of individuals exposed to small doses that can be transmitted to the offspring [74], paving the way to transgenerational carcinogenesis [54]. It has been shown that these effects are greater if the father or both parents are exposed [75]. The data about the major effect on the offspring when the male parent is exposed are in perfect accordance with the notion of the greater sensitivity of paternal (immature) gametes to chemical and physical (epi)genotoxic agents: which is an indirect confirmation of the epidemiological data, for a long time disputed, concerning the increase of leukemia in children of the male parent resident in the vicinity of nuclear facilities [76]. It is nonsense to oppose this evidence the data found in the offspring of people exposed for occupational reasons, due to the different ways of exposure, which are mostly external versus internal [77].

Also, bystander effects have been recently studied on the light of epigenetics. It is highly reliable that epigenetic alterations can be triggered by the bystander signal(s) and may play a significant role in the outcomes of IR in nonirradiated cells and tissues. Indeed, besides the induction of DNA damage, also global DNA methylation, cell proliferation and apoptosis both in exposed and bystander spleen tissue of male and female mice of two different strains (C57BL/6 and BALB/c) were observed. In this study the bystander effects were studied in the spleen of animals subjected to 1 Gy of X-ray, through only skull exposure, while the rest of the animal body was protected by a lead shield (the same used for human body protection in diagnostic radiology) [78].

Moreover, paternal X-ray irradiation leads to a significant accumulation of DNA damage, loss of global methylation and altered global expression of miRNAs in the paternal germline, also influencing global DNA methylation in bone marrow and thymus in unexposed offspring [75,79]. Therefore, in many cell/tissue cultures and in in vivo experimental studies aberrant DNA methylation, as well as altered miRNA levels, involved in epigenetic regulation, besides a series of other factors (such as free radicals, cytokines...), have been detected in distant tissues shortly after irradiation. This effect can persist for a long time after exposure (for a review see [80]), which can have significant implications for a better understanding of radiation carcinogenesis as well as of diagnostic radiological procedures and radiotherapy [81].

One of the main consequences of the adoption of an epigenetic model in IR carcinogenesis derives from the persistence and accumulation of epigenetic changes: while the fate of most genetic mutations is to be repaired or lost (along with the cells weakened because transformed), genomic instability is, in fact, destined to grow over time, both in subsequent cell generations, and in the offspring of exposed people. This applies, a fortiori, for individuals exposed in utero and for trans-generational effects resulting from the exposure of germ cells. In this regard experimental data are still few [74,82], because radiosensitivity varies greatly between species and in relation to exposure scenarios and the radiation history of the organisms and their parents. 

On the other hand, the adoption of an epigenetic model could be useful to better explicate the controversial data concerning the limited effects of massive exposure to IR on subsequent generations, as in the case of Hiroshima and Nagasaki survivors. Indeed, while the gametes genetically damaged in the case of exposure to high doses are largely eliminated, on the contrary, the epigenetically marked gametes do not have such a competitive handicap. Moreover, the epigenetic modifications will be accumulating from one generation to another. Therefore, the offspring of individuals chronically exposed to relatively lower doses of radiation will paradoxically suffer the most severe effects and will transmit them to their descendants [74]. This also means that even with regard to Chernobyl, the main consequences may become evident only in the coming decades. Also, the data recently obtained about the increase in childhood leukemia close to the nuclear power plants could be easily explained [82,83]. 

## 5. Conclusions

Large-scale projects of genome sequencing allowed significant progress in identifying the mutational profile of cancers. Some mutational signatures have been linked to the action of endogenous processes or environmental exposures; e.g. tobacco smoke, ultraviolet light, aflatoxins, aristolochic acid, and IR [84]. Recent studies documented the existence and persistence of cancer epigenetic signatures related to environmental exposures, such as cigarette smoking; however, until now this has been limited to one epigenetic mark (i.e., DNA methylation), it is likely that in the future the analysis of mutational and epigenetic signatures in experimental studies as well as in epidemiological studies could represent a very promising area of research on exposure to IR [85]. Considering such emerging evidence, there is the need for a changing paradigm in the interpretation of the mechanisms leading to cellular damage after exposure to IR, moving from a traditional paradigm based on the “stochastic breakage” of one or both strands of the DNA double helix induced only by high dose exposures towards an epigenetic model which could also explain the transgenerational effects of mild and persistent irradiation [54,74].
